# Soil ionomic and enzymatic responses and correlations to fertilizations amended with and without organic fertilizer in long-term experiments

**DOI:** 10.1038/srep24559

**Published:** 2016-04-15

**Authors:** Xumeng Feng, Ning Ling, Huan Chen, Chen Zhu, Yinghua Duan, Chang Peng, Guanghui Yu, Wei Ran, Qirong Shen, Shiwei Guo

**Affiliations:** 1 Jiangsu Key Laboratory for Solid Organic Waste Utilization, Nanjing Agricultural University, Nanjing, 210095, China; 2 Crop Research Institute, Anhui Academy of Agricultural Science, Hefei, 230031, China; 3 Institute of Agricultural Resources and Regional Planning, Chinese Academy of Agricultural Sciences, Beijing, 100081, China; 4 Agriculture Environment and Resources Center, Jilin Academy of Agricultural Sciences, Changchun, 130033, China

## Abstract

To investigate potential interactions between the soil ionome and enzyme activities affected by fertilization with or without organic fertilizer, soil samples were collected from four long-term experiments over China. Irrespective of variable interactions, fertilization type was the major factor impacting soil ionomic behavior and accounted for 15.14% of the overall impact. Sampling site was the major factor affecting soil enzymatic profile and accounted for 34.25% of the overall impact. The availabilities of Pb, La, Ni, Co, Fe and Al were significantly higher in soil with only chemical fertilizer than the soil with organic amendment. Most of the soil enzyme activities, including α-glucosidase activity, were significantly activated by organic amendment. Network analysis between the soil ionome and the soil enzyme activities was more complex in the organic-amended soils than in the chemical fertilized soils, whereas the network analysis among the soil ions was less complex with organic amendment. Moreover, α-glucosidase was revealed to generally harbor more corrections with the soil ionic availabilities in network. We concluded that some of the soil enzymes activated by organic input can make the soil more vigorous and stable and that the α-glucosidase revealed by this analysis might help stabilize the soil ion availability.

In the last 50 years, China have remarkable growth in agricultural production. This has created the so called “Miracle in China” with 7% of the world’s arable land feeding 22% of the world’s population. The intensification of crop production over the last 50 years has been achieved through the use of modern high-yielding varieties, and major benefits have been realized by using chemical fertilizers^1^. China is currently the world’s largest consumer of mineral fertilizer, the consumption of which has increased almost linearly because farmers prefer to use greater amounts of chemical fertilizers to attain higher yields. Unfortunately, this high increment of mineral fertilizer consumption coexists with extremely low fertilizer nutrient use efficiency, which is attributed to widespread environmental damages^2^,^3^. Therefore, government is planning to reduce the consumption of chemical fertilizers and pesticides in future years while maintaining high crop production. The partial replacement of chemical fertilizers with organic fertilizers is an alternative way to achieve this goal.

Since China has large livestock and poultry breeding industries^4^, the use of the organic wastes can reduce chemical fertilizer input easily, and thus maintain high crop production to meet the food requirement of the large population and improve the soil quality^5^,^6^. The influence of long-term organic fertilizer applications on soil physical and chemical properties^7^,^8^, soil fertility^9^, and crop yield^10^ has been studied. In recent years, concerns about the quality of fertilizer products have extended beyond nutrient content to include the potential presence of non-nutrient toxic substances, such as heavy metals, and their interactions with the agricultural environment. Available information remains limited about the long-term influence of mineral fertilizers and organic manure on the availability of soil ions, particularly the heavy metals. Both short- or long-term exposure to toxic metals can reduce microbial diversity and activities in soil^11^,^12^, through effects on both enzyme activities and cellular macromolecules via free radical formation. Therefore, it is also important to investigate the response of soil ions and enzyme activities to fertilization with and without organic fertilizer and clarify the correlations from long-term field experiments.

In this study, we used the soil ionome as the research objective and also investigated 11 types of enzymes that were involved in the C, N, P, and S transformation in soil. Because the soil ionomic and enzymatic response to organic amendment could be a long process, any convincible evidence can surely be obtained via long-term experiments. We hypothesized that some of methods in the repertory of statistical approaches so far available to ecologists, including network analysis, should provide ideal way to exhibit the varied correlations between the soil ionome and the soil enzyme activities and among the soil ions as suffered by different long-term fertilization. Thus, in this study, soil samples, subjected to chemical fertilization amended with (COF) or without (CF) organic fertilizer, were collected from 4 long-term field experiments, all performed over 23 years and located in different provinces of China. Exploring the large soil datasets generated using modern detection technologies requires new analytical approaches to move beyond the basic inventory descriptions of the variations of soil parameters, therefore a novel analysis was also conducted to decipher the soil properties interactions from a new cognitive angle, aiming to clarify how the soil ionome and enzyme activities respond to the organic amendment versus only chemical fertilizer application and to decipher the correlations between the soil ionome and enzymes and between the available ions in the soils.

## Results

### Effects of pH, sampling site, fertilization and their interactions on soil ionomic profile

**Figure 1:**
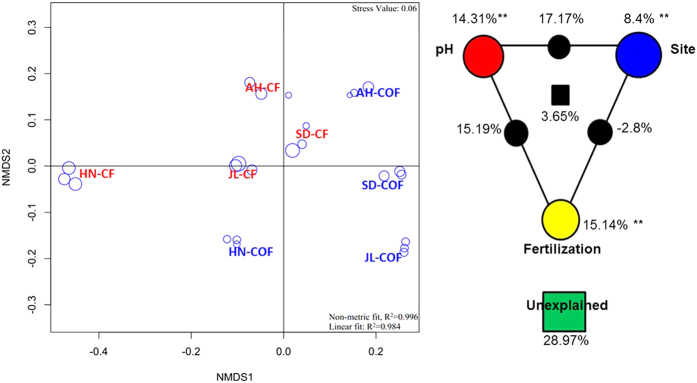
NMDS plot (left) of Bray–Curtis dissimilarity matrix of ionomic concentration data among 24 samples from long-term experiments and VPA map (right) of the effects of pH, experimental site, fertilization, and their interactions on the soil ionome. In the NMDS plot, stress values and Shepard R^2^ values are shown in the upper right and the lower right of the plot, respectively. The bubble size indicated the goodness of fit, and poorly fitted sample have larger bubbles. JL, SD, AH and HN indicate the samples collected from the long-term experiments at Jilin Province, Shandong Province, Anhui Province and Hunan Province of China, respectively. CF means the treatment applied with only chemical fertilizer, and COF means the treatment applied with both organic and inorganic fertilizer. The treatments labeled in blue are collected from the COF treatment, and the treatments labeled in red are collected from the CF treatment. The three bubbles closest to the treatment label stand for the three replicates of each treatment obtained from the same long-term experiment. In the VPA map, circles on the edges of the triangle show the percentage of variation explained by each factor alone. The percentage of variation explained by interactions between two or three of the factors is shown as black circles on the sides and as a square at the center of the triangle. The unexplained variation is depicted in the square at the bottom. **Indicate significance at P < 0.01.

To visualize the differences in the soil ionomic profile between the CF and COF samples, the ionomic concentration profiles were used to prepare a Bray–Curtis dissimilarity matrix, which was coordinated into two dimensions by nonmetric multidimensional scaling (NMDS) (Fig. 1). The stress value of the NMDS was 0.06, and the R^2^ values of the non-linear and linear regressions of the NMDS distances on the originals were 0.996 and 0.984, respectively, and these results indicated that well-fitted ordination was obtained. The samples were grouped according to the sampling site and type of fertilization and were separated mainly by the fertilization type along the first axis and also divided by sites along the second axis.

To quantify the relative contributions of pH, site and fertilization to the soil ionomic profile, variance partitioning analysis (VPA) was carried out (Fig. 1). The variation in the ionomic profile structure was partitioned among pH, site and fertilization type and the interactions among them. These variables explained 71.03% of the observed variation, leaving 28.97% of the variation unexplained (Fig. 1). For these independent variables, fertilization type had the major impact on the soil ionomic profile, which accounted for 15.14%, followed by pH and site, which accounted for 14.31% and 8.4%, respectively. The interactions between pH and site and pH and fertilization explained 17.17% and 15.19%, respectively, of the variation. A negative impact was found between site and fertilization type on the soil ionomic profile, the interaction of which accounted for only −2.8% of the variation.

### Effects of pH, sampling site, fertilization and their interactions on soil enzymatic profile

**Figure 2:**
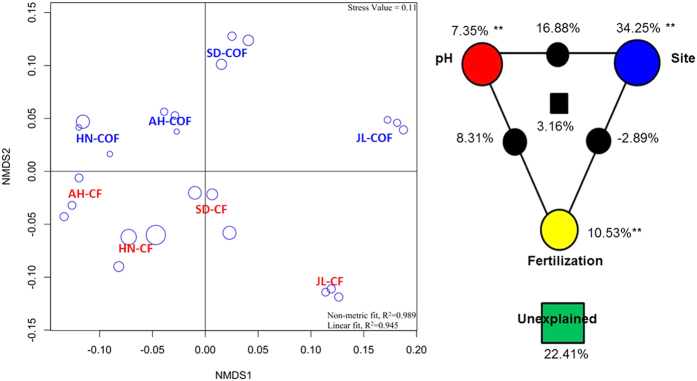
NMDS plot (left) of Bray–Curtis dissimilarity matrix of enzyme activity data among 24 samples from long-term experiments and a VPA map (right) of the effects of pH, experimental site, fertilization, and their interactions on the soil enzymes. In the NMDS plot, stress values and Shepard R^2^ values are shown in the upper right and the lower right of the plot, respectively. The bubble size indicated the goodness of fit, and poorly fitted sample have larger bubbles. JL, SD, AH and HN indicate the samples collected from the long-term experiments at Jilin Province, Shandong Province, Anhui Province and Hunan Province of China, respectively. CF means the treatment applied with only chemical fertilizer, and COF means the treatment applied with both organic and inorganic fertilizer. The treatments labeled in blue are collected from the COF treatment, and the treatments labeled in red are collected from the CF treatment. The three bubbles closest to the treatment label stand for the three replicates of each treatment obtained from the same long-term experiment. In the VPA map, circles on the edges of the triangle show the percentage of variation explained by each factor alone. The percentage of variation explained by interactions between two or three of the factors is shown as black circles on the sides and as a square at the center of the triangle. The unexplained variation is depicted in the square at the bottom. **Indicate significance at P < 0.01.

A NMDS plot was also drawn to visualize the differences in soil enzymatic profile between the CF and COF samples (Fig. 2). High goodness-of-fit was also observed in this plot; the stress value of the NMDS was 0.11, and the R^2^ values of the non-linear and linear regressions of the NMDS distances on the originals were 0.989 and 0.945, respectively. The samples were grouped according to site and fertilization type and were primarily separated by the site along the first axis and by the fertilization type along the second axis.

VPA was used to determine the relative contributions of pH, site and fertilization to the soil enzymatic activities (Fig. 2). These variables explained 77.59% of the observed variation, leaving 22.41% of the variation unexplained. For these variables, site made the major impact on the soil enzymatic profile, accounting for 34.25%, followed by fertilization and pH, which accounted for 10.53% and 7.35%, respectively. In the interactions, the one that explained most of the variation was found between pH and site, the partition of which accounted for 16.88%. The interactions between pH and fertilization accounted for only 8.31% of the variation. The interaction between site and fertilization negatively impacted the soil enzymatic profile and accounted for −2.89% of the variation.

### Response ratio between organic and non-organic amendment

**Figure 3:**
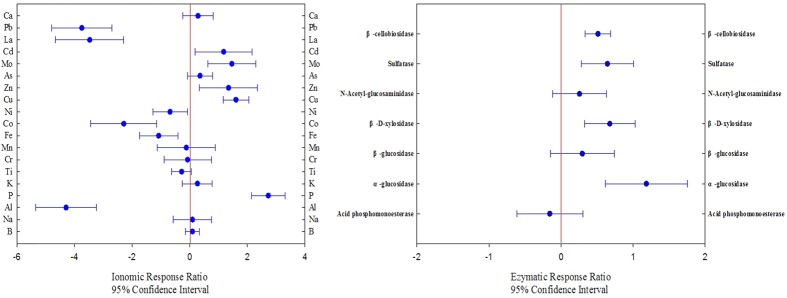
Ionomic and enzymatic response ratios in soils under long-term organic amendment relative to long-term chemical fertilizer input only. Blue dots refer to the response ratio, and the error bars represent the 95% confidence intervals. Confidence intervals overlapping with the vertical line drawn at zero indicate no significant effect.

The response ratios (RR) of the soil exchangeable ions and soil enzyme activities to organic amendment were evaluated at 95% confidence intervals (Fig. 3). The availability of Cd, Mo, Zn, Cu and P significantly increased under long-term organic fertilizer amendment compared with only the long-term application of chemical fertilizer. Compared with COF, the exchangeabilities of Pb, La, Ni, Co, Fe and Al in CF were significantly higher no matter in which sampling site. The fertilization displayed the greatest impacts on soil available Al and P, which showed the lowest (−1.21) and highest (1.01) values in the response ratio respectively. Most of the soil enzyme activities were promoted by the long-term application of organic fertilizer. Indeed, the activities of α-glucosidase, β-D-xylosidase, sulfatase and β-cellobiosidase were significantly activated by the long-term organic fertilizer amendment.

### Network analysis between soil enzyme profile and soil ionomic profile

**Figure 4:**
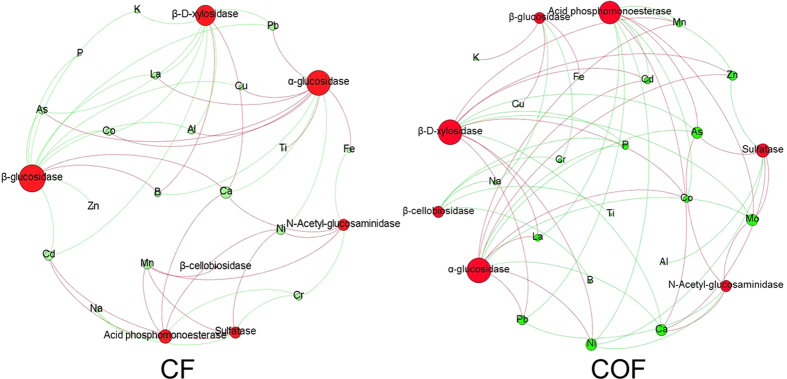
Network of CF and COF based on correlation analysis between ionomic and enzymatic profiles. Red nodes indicate enzymatic affiliation, and blue nodes indicate ionomic categories. A connection indicates a significant (P < 0.05) correlation. The red and green edges stand for negative and positive correlations, respectively. The size of each node is proportional to the number of connections (that is, degree). CF means the treatment applied with only chemical fertilizer, and COF means the treatment applied with both organic and inorganic fertilizer.

Network inference was employed to explore co-occurrence patterns between the soil enzyme activity profile and the soil ionomic profile (Fig. 4, Table S1). In general, there were fewer correlations in the CF than in the COF soils. In the CF soils, the network had 25 nodes and 46 edges (26 positive correlations), the modularity was 0.344 with 2 communities, and the α- and β-glucosidase activities were the most active factors. For the COF soils, the network had 26 nodes and 53 edges (27 positive correlations), the modularity was 0.295 with 3 communities, and the α-glucosidase, acid phosphomonoesterase and β-D-xylosidase activates were the most active factors. In the networks, we selected α-glucosidase activity as the generalist because it had the most connections in both CF and COF. Most of the connections (72.7% in both CF and COF) of α-glucosidase were negative, which indicated that it may provide a certain contribution on stabilizing the ion availability.

### Network analysis within the soil ionome

**Figure 5:**
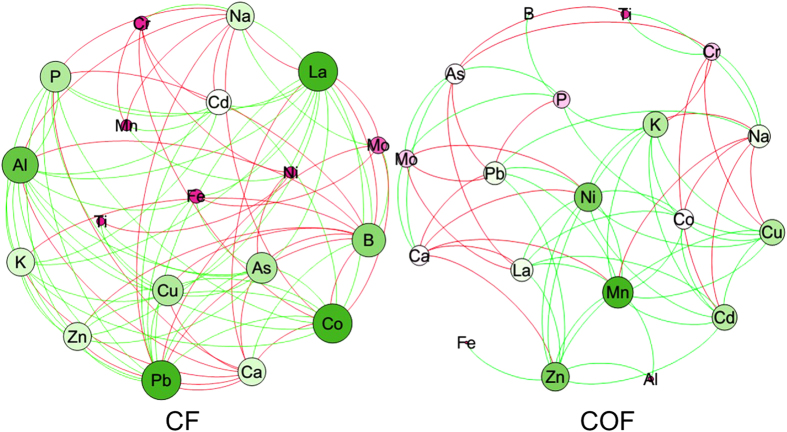
Network of CF and COF based on correlation analysis among ionomic profiles. Nodes indicate ionomic categories. A connection indicates a significant (P < 0.05) correlation. The red and green edges stand for negative and positive correlations, respectively. The size of each node is proportional to the number of connections (that is, degree). CF means the treatment applied with only chemical fertilizer, and COF means the treatment applied with both organic and inorganic fertilizer.

We further explored co-occurrence patterns within the soil ionome by using network inference based on Pearson’s significant correlations (Fig. 5, Table S1). The network analysis was markedly different for the CF and COF samples, and the connections in the CF samples were much more complicated than were those in the COF samples. In the CF, the network had 19 nodes and 88 edges (53 positive correlations), with 4.632 average degrees (connections). For the COF, the network had 19 nodes and 64 edges (43 positive correlations), with 3.368 average degrees (connections). More active ions (with high degrees), such as Pb, Co, Al, Na, Ca, As, La, B, Cu, K, P and Zn, were found in CF, the degrees of which were greater than 10. In the COF, only Mn, Ni and Zn were found to have the degree greater than 10. In this network, Zn was active in both the CF and COF samples. Most of the positive connections of Zn, which were 80% and 90% in the CF and COF samples, respectively, were found, which indicated that Zn could play a certain role in inter-activating ion availability.

## Discussion

Unraveling the relative importance of fertilization patterns versus soil types in the full-scale evaluation of soil fertility pose significant challenges. Data currently available are mostly based on the simple comparison of soil properties in long-term fertilizations^13^,^14^. Moreover, organic waste is also recognized to be a significant source of contaminants in depositions in soils^15^. While these previous works have provided great insights into the effects of fertilizations on individual soil factors, the classic statistical way used in these works have a limited ability to differentiate the combined effects of soil properties that occur in agricultural ecosystems. Thus, in our study, the samples from long-term experiments (Table 1) were analyzed in depth and a series of advanced statistical analyses were performed. The ordination of the soil ionomic and enzymatic profiles were generated and revealed a clear separation between the CF and COF samples (Figs 1 and 2), which indicates a selective change in the ion availability and enzyme activities under the organic amendment compared with long-term chemical fertilizer-treated soils, as noted in other studies^16^,^17^,^18^.

Many studies have shown that environmental factors directly or indirectly shape ion availability and enzyme activities^16^,^19^. Soil pH is widely accepted as a dominant factor that regulates soil nutrient bioavailability, microbial community composition, and plant primary productivity^20^,^21^. We also found that the soil treated long-term with chemical fertilizer only was severely acidified (Table S2). This result coincided with evidence that the overuse of chemical fertilizer contributes substantially to regional soil acidification in China^2^ and agreed with the observation that a significant increase in soil pH can result from organic amendments^22^. To differentiate the pH between treatments in the same sample site, we considered pH to be an independent factor on VPA (Figs 1 and 2). In the present study, most of the variation in the ionomic profile can be explained by the interaction between different fertilization patterns, and pH is the second greatest impact on ion behavior (14.31%). These results confirmed the previously reported findings that fertilization impacts the soil pH^2^ and that their interactions acted on the availability of soil ions^23^. Fertilization had the second greatest impact on ionomic variation (15.14%), which was consistent with a report that organic input could have different effects on metal behavior depending on the metal, the soil and the characteristics of the organic matter^23^. The organic matter of manure can be used to immobilize and bind special ions in the soil. In the VPA, the primary factor that affected soil enzyme activities was site (Fig. 2). The same site shared the same soil type, climate, crop regimes, and irrigation, among other factors. The enzyme activities were attributed to soil microbial communities. Thus our results also indicated that the soil microbial function assembly was predominantly impacted by the geographical location, which shares many of the same contexts, including soil type, climate, and crop regimes^24^. In the VPAs of both the ionomic and enzymatic profiles, a negative interaction was detected between site and fertilization. The result indicated that fertilization could have an opposite influence from that of the soil type, climate, and crop regimes on soil ion availability and enzyme activity. It showed that appropriate fertilization could manipulate the defects of soil nutrients availability and microbial activity that were caused by the original soil quality.

The availabilities of ions and the activities of enzymes in the soil produce various responses to fertilization (Fig. 3). Organic fertilizers provide more stable humic substances with large surface areas and components of long-chain fatty acids, aliphatic alcohols, and linear hydrocarbons, which account for the exchangeability of minerals^4^,^16^,^25^. At the same time, the organic input offers an organic substrate for soil microbes and activates their functions^17^. Compared with the samples that had no organic input, the P availability showed the largest increase among the tested ions following treatment with organic fertilizer (Fig. 3). Further, the available Zn, Cu, Mo and Cd were significantly increased by the long-term application of organic fertilizer.

Most organic fertilizer in China originates from farmyard manure. Due to the use of feed additives, livestock and poultry manures from factory farms contain high heavy metal concentrations, particularly the heavy metals Cu and Zn^26^. Additionally, manure is rich in Cd, which might accumulate in the soil^16^. Compared with long-term organic amendment, applying only the chemical fertilizer to the soil resulted in the higher availability of Pb, La, Ni, Co, Fe and Al, most of which are categorized as heavy metals and bear potential harm to plants, animals, human beings, and microorganisms. The most significant impact of long-term chemical fertilization on ion availability is due to Al, which is well known as an acidic ion. Apparently, most of the activated cations by long-term chemical fertilization are available at slight acidic conditions and precipitated at alkaline pH values. Soils are strongly buffered by ion exchange reactions, by the weathering of soil minerals, and (in the acidic range) by interactions with Al and Fe^27^. Therefore, the significantly increased availability of Al, Fe and so on could have a certain contribution or relationship to soil acidification because of long-term chemical fertilization. Moreover, the distinct profiles of soil potential enzyme activities between CF and COF indicated unique potential metabolic capacities in the soils. Many studies have indicated that soil enzyme activities are often enhanced by organic amendments and are significantly correlated with soil organic carbon content^28^. In our study, most of the determined enzyme activities were significantly increased by organic amendment, particularly for α-glucosidase, which was the enzyme that exhibited the highest increment (Fig. 3). Greater enzyme activities is the stronger fundamental driver of carbon and nutrient recycling in the soil and, in turn, will induce higher mineralization and decomposition of organic matter. The product and intermediate in the process of mineralization and decomposition can be used to immobilize and bind heavy metal ions in the soil, thereby affecting ion availability. Especially, the dissolved organic matter (DOM), a non-homogeneous produced by the decomposition of organic waste or humus, is composed of macromolecular humus and small molecular non-humus materials and can strongly bind heavy metals in the environment to influence the solubility and mobility of these metals. Thus, DOM can serve as a carrier for metals and impact metal availability. However, available ions can indirectly affect soil enzymatic activities by altering the microbial communities that synthesize enzymes, and their mode varies with the enzyme type or can directly inactivate the enzyme^12^,^29^. Hence, it is reasonable to deduce that an interaction can occur between soil enzymes and the soil ionome. However, there are many uncertainties about exactly how the interaction occurs.

Network analyses were conducted to gain a more integrated understanding of the soil ionomic and enzymatic traits and to compare the complexity of the networks operating between the soil ionome and the soil enzymes (Fig. 4) and between the soil ions (Fig. 5). Our analysis was constructed using all significantly positive and negative correlations. In this study, there were fewer correlations between the soil ionome and the soil enzymes in the CF samples than in the COF samples (Fig. 4). Considering that the activity of most soil enzymes can be activated by organic amendment, the soil could become more vigorous, which may result in more interactions between the ions and enzymes. Organic input can stabilize the exchangeability of soil ions by regulating the pH and adsorption^4^,^30^; and thus, a less complex network between the soil ions in COF would be expected (Fig. 5). These results also indicated that organic amendment can improve soil enzyme activity and stabilize soil ion availability. Moreover, in the CF samples, more ions with corrections greater than 10 were detected, including Na and Ca, and most of the corrections of Na and Ca were negative. This finding demonstrated that saline soil and calcimorphic soil with long-term chemical fertilizer application rather than organic amendment are unacceptable, because these soils have high background values of Na and Ca contents which could lead to serious variation in the ion network if the soil is treated long-term only with inorganic fertilizer. Zn exclusively presented a high number of correlations, which interestingly belonged to both the CF and COF networks. These data could point to an important role of Zn in mediating soil ion availability by keeping important connections on a larger scale with other groups and displaying certain important functional traits. The α-glucosidase was also generally found in both the CF and COF networks and negatively correlated with the ion availability mostly (Fig. 4). In our results, the α-glucosidase activity was activated (Fig. 3), and the network of the soil ionome became more stabilized by organic amendment (Fig. 5). Therefore, it is possible to deduce that organic amendment can stabilize soil ion interaction and improve the soil buffering capacity, which might partially be attributed to the activated α-glucosidase activity. To the best of our knowledge, this is the first study to utilize the novel approach of soil ionomics with mutual information to simultaneously investigate the single ion and multiple ions that were represented by ion networks and the associations with soil enzymes. The next logical step is to go beyond merely describing the patterns revealed by the network analysis and design more focused experiments to understand the mechanisms producing patterns of α-glucosidase activity and soil stability.

In summary, our results suggest that organic amendment can regulate soil ionome and soil enzyme activities, which result in benefits to the soil nutrient supply state and plant heavy metal content. The novel analysis approach also deciphered soil vitality as an aspect of the network between the soil ionome and the soil enzymes and clarified the manipulation power of organic fertilizer and other environmental variables in shaping soil functions. The α-glucosidase was exposed by the analysis and might provide a certain contribution to stabilizing soil ion availability. Further analysis is needed to better understand the mechanisms by which α-glucosidase mediates soil ion availability through activation by organic input, whereby the study of the role of organic fertilizer in shaping soil functions is of prime importance.

## Methods

### Field sites and sample collection

Four long-term fixed fertilization experiments were selected throughout the major grain producing areas of China. As shown in Table 1, the first long-term experiment was located in Jilin Province (JL) and was initiated in 1990; the climatic conditions, cropping system and physical and chemical characteristics of the initial field soil were all introduced in our previous paper^17^. The second long-term experiment was located in Shandong Province (SD) and has been performed since 1978; detailed information about the climate, cropping system and physical and chemical characteristics of the initial field soil in this experiment was described by Song *et al.*^31^. The third long-term experiment was set in Anhui Province (AH), which is located in the Yangliu experimental base of Anhui agricultural academy and was conducted starting in 1981 with wheat-maize rotation. The primary soil chemical and physical properties of the AH long-term experiment were pH (H_2_O) of 7.6, 10.22 g · kg^−1^ organic matter, 0.78 g · kg^−1^ total N, and 2.5 g · kg^−1^ of available P. The fourth long-term experiment was carried out starting in 1990 at Hunan Province (HN), the basic status of which was published previously^32^,^33^. According to the China Soil Classification System^34^, the soil great groups of each experimental site are listed in Table 1.

In every long-term experiment, two treated soils were collected: the soil applied only with chemical N, P, and K fertilizers (CF) and the soil applied with chemical fertilizers plus organic fertilizer (COF). The chemical properties of sampled soil and the fertilizer application rate of the treatments in each long-term experiment are listed in Table S2. Three replicates, which were collected from three separated plots, were sampled in each treatment at each experimental site. Each sample was composited with 10 soil cores (5 cm diameter) from each plot at a depth of 0–20 cm. All soil samples were collected from the four long-term experiments (2 treatments *3 replicates *4 experiments = 24 samples in total) at the fallow stage, post-harvest but before the next season’s crop planting in 2013–2014. In order to maximally evaluate the long-term effect of the fertilization and avoid the nonuniformity of moisture between samples from different sites, all the soil samples were air dried for about 7 days to maintain ~15% soil water content, and then sieved through a 1.0 mm sieve in preparation for the analyses of exchangeable ions and soil enzyme activities.

### Soil ionomic analyses

The exchangeable ions, B, Na, Al, P, K, Ti, Cr, Mn, Fe, Co, Ni, Cu, Zn, As, Mo, Cd, La, Pb and Ca, were extracted from each sample. The available P was extracted with sodium bicarbonate and then determined by the molybdenum-blue method^6^. The other ions were extracted by 1 M Mg(NO_3_)_2_ (pH7, Soil: Mg(NO_3_)_2_ = 2.5:10) after shaking for 2 h at 25 °C. The soil extracts were filtered, and the ionomic concentrations of soil extracts were determined by inductively coupled plasma mass spectrometry (ICP-MS, Perkin Elmer Nexion 300×) in the He gas collision mode to minimize polyatomic interferences.

### Enzyme activities analysis

The potential activities of 7 enzymes involved in C, N, P, and S cycling, i.e., acid phosphomonoesterase, sulfatase, β-glucosidase, β-cellobiosidase, N-acetyl-glucosaminidase, β-xylosidase, and α-glucosidase, of soil samples were measured using 4-methylumbelliferyl-esters as substrates, producing fluorescent 4-methylumbeliferone (MUF) after hydrolysis^35^. A 96-well microplate was used for these analysis as described by Deng *et al.*^36^. The fluorescence intensity was determined by a microplate fluorometer (Scientific Fluoroskan Ascent FL, Thermo) with 365 nm excitation and 450 nm emission filters. The enzyme activities were expressed as nanomoles per hour per gram of soil (Table S3).

### Data analysis

Nonmetric multidimensional scaling (NMDS) plots were used to visualize the profile variations among samples, using the ionomic and enzymatic concentration matrix. The plots were generated from Bray–Curtis dissimilarity index matrices of the 24 samples. Variance partitioning analysis (VPA) was also used to determine the contributions of pH, locations, and fertilization and the interactions among them on the variation in ionome and enzyme activities with Hellinger-transformed data. NMDS and VPA were performed by R (version 2.15.0) with the vegan packages^37^.

The effects of organic amendment on the ionome and enzyme activities across the experiments were analyzed using the response ratio (RR) as a common effect size metric^38^. To quantify simple two-group experimental designs, the calculation of RR is straightforward:


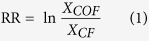


Here, R is the natural-log proportional change in the means (X) of the target variable of CF (X_CF_) and OF (X_COF_), respectively. The analysis was performed on a spreadsheet using a random effect model according to Geisseler and Scow^20^.

The analysis, when pooling RR from multiple studies, also assigns a weight to each R that is inversely proportional to its sampling variance:


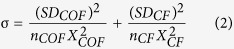


where SD and n are the standard deviation and sample size (n = 12 in this study) of X_COF_ and X_CF_, respectively^39^. The 95% confidence interval (CI) for the response ratio is:


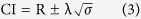


λ = 1.96 for 95% CI. The difference is significant only if 95% of a response variable does not overlap with zero^40^. A positive response ratio indicates an increase in soil exchangeable ion concentrations and soil enzyme activities under long-term organic fertilizer amendment relative to the set of soil ions and enzyme activities of only chemical fertilization, whereas a negative response ratio indicates the opposite.

Network analyses were performed to better understand the relationships between enzymes and ions and between ions within the long-term experiments. To analyze the networks, we calculated all possible Pearson’s correlation coefficients. To filter the data for reduced network complexity, we considered high correlations with a cutoff at r > 0.58 and a statistically significant P-value < 0.05, accounting for all replicates. The nodes in the reconstructed network represent ionomic and enzymatic groups, and the edges represent high and significant correlations between nodes. The network graphs were made based on a set of measures, as average node connectivity, average path length, diameter, and cumulative degree distribution^41^. Statistical analyses were carried out in the R software package (http://www.r-project.org/) and network visualization with the interactive platform Gephi^42^.

## Additional Information

**How to cite this article**: Feng, X. *et al.* Soil ionomic and enzymatic responses and correlations to fertilizations amended with and without organic fertilizer in long-term experiments. *Sci. Rep.* **6**, 24559; doi: 10.1038/srep24559 (2016).

## Supplementary Material

Supplementary Information

## Figures and Tables

**Table 1:** The location, soil great group and initial time of the long-term field experiments in China used in this study.

Sampling site abbreviation	Coordinates	Location	Soil great group	Experiment initiation year
JL	124.8°E,	Gongzhulin, Jilin Province	Black Soils	1990
	43.5°N			
SD	120.7°E	Laiyang, Shandong Province	Fluvo-aquic Soils	1978
	36.9°N			
AH	116.75°E	Suixi, Anhui Province	Lime Concretion Black Soils	1981
	33.62°N			
HN	111.87°E	Qiyang, Hunan Province	Red Soils	1990
	26.75°N			
