# Supplementary material for: Soil ionomic and enzymatic responses and correlations to fertilizations amended with and without organic fertilizer in long-term experiments
Source: Sci Rep. 2016 Apr 15;6:24559. doi: 10.1038/srep24559 (PMC4832195; doi:10.1038/srep24559)
Supplement: Supplementary Information [file srep24559-s1.doc]

**Soil** **ionomic and** **enzymatic responses and correlations to fertilizations amended with and without organic fertilizer in long-term experiments**

Xumeng Feng^§, 1^, Ning Ling ^§, *, 1^, Huan Chen^2^, Chen Zhu^1^, Yinghua Duan^3^, Chang Peng^4^, Guanghui Yu^1^, Wei Ran^1^, Qirong Shen^1^, Shiwei Guo^*, 1^

1, Jiangsu Key Laboratory for Solid Organic Waste Utilization, Nanjing Agricultural University, Nanjing, 210095, China.

2, Crop Research Institute, Anhui Academy of Agricultural Science, Hefei, 230031, China.

3, Institute of Agricultural Resources and Regional Planning, Chinese Academy of Agricultural Sciences, Beijing, 100081, China.

4, Agriculture Environment and Resources Center，Jilin Academy of Agricultural Sciences, Changchun, 130033, China.

**^§^Xumeng Feng and Ning Ling contribute equally to this paper.**

***To whom the correspondence should be addressed.**

1, Dr. Ning Ling

E-mail: [nling@njau.edu.cn](mailto:nling@njau.edu.cn)

2, Prof. Dr. Shiwei Guo

E-mail: sguo@njau.edu.cn

Table S1 The description of the networks.

|  | **Total nodes** | **Total edges** | **Positive correlations** | **Diameter** | **Clustering coefficient** | **Modularity** | **Number of Communities** |
| --- | --- | --- | --- | --- | --- | --- | --- |
| **Between ionome and enzymes** | | | | | | | |
| **CF** | 25 | 46 | 26 | 5 | 0 | 0.344 | 2 |
| **COF** | 26 | 53 | 27 | 8 | 0 | 0.295 | 3 |
| **Between ions** | | | | | | | |
| **CF** | 19 | 88 | 53 | 3 | 0.725 | 0.135 | 3 |
| **COF** | 19 | 64 | 43 | 4 | 0.595 | 0.195 | 3 |

CF means the treatment applied with only chemical fertilizer, and COF means the treatment applied with both organic and inorganic fertilizer.

Table S2 The chemical properties of sampled soil and the fertilizer application rate of treatments in each Long-term experiments

| **Long-term experiment site** | **Treatment** | **pH when Sampling** | **TC (%) when Sampling** | **TN (%) when Sampling** | **C/N when Sampling** | **Inorganic fertilizer rate [kg/(ha*season)]** | | | **Organic fertilizer rate**  **[kg/(ha*season)]** | |
| --- | --- | --- | --- | --- | --- | --- | --- | --- | --- | --- |
|  |  |  |  |  |  | **N** | **P_2_O_5_** | **K_2_O** |  |  |
| **JL** | CF | 5.96±0.09 | 1.32±0.03 | 0.14±0.01 | 9.52±0.23 | 165 | 82.5 | 82.5 | | - |
|  | COF | 7.59±0.07 | 2.46±0.02 | 0.25±0.01 | 9.84±0.05 | 165 | 82.5 | 82.5 | | 6000 |
| **SD** | CF | 6.74±0.10 | 0.69±0.05 | 0.08±0.01 | 8.84±0.27 | 276 | 90 | 135 | | - |
|  | COF | 7.26±0.02 | 1.49±0.01 | 0.16±0.01 | 9.07±0.27 | 276 | - | - | | 6000 |
| **AH** | CF | 5.75±0.13 | 1.18±0.03 | 0.14±0.01 | 8.35±0.06 | 262.5 | 105 | 105 | | - |
|  | COF | 7.29±0.22 | 1.57±0.02 | 0.15±0.01 | 10.60±0.18 | 132 | 52.5 | 52.5 | | 3750 |
| **HN** | CF | 4.12±0.15 | 1.20±0.05 | 0.14±0.02 | 8.47±1.08 | 300 | 120 | 120 | | - |
|  | COF | 5.79±0.18 | 1.60±0.02 | 0.18±0.01 | 8.84±0.23 | 300 | 120 | 120 | | 3000 |

TC: Total carbon; TN: Total nitrogen; C/N: the ratio of carbon to nitrogen. The values are expressed as means ± S.E. JL: the long-term experiment conducted in Jilin Province of China; SD: the long-term experiment conducted in Shandong Province of China; AH: the long-term experiment conducted in Anhui Province of China; HN: the long-term experiment conducted in Hunan Province of China. CF means the treatment applied with only chemical fertilizer, and COF means the treatment applied with both organic and inorganic fertilizer.

  Table S3 The enzymes activities (nmol·h^-1^·g^-1^) of sampled soils in each Long-term experiments

| **Long-term experiment site** | **Treatment** | **Acid phosphomonoesterase** | **α-glucosidase** | **β-glucosidase** | **β-D-xylosidase** | **N-Acetyl-glucosaminidase** | **Sulfatase** | **β-cellobiosidase** |
| --- | --- | --- | --- | --- | --- | --- | --- | --- |
| **JL** | CF | 202.64±0.72 | 19.61±0.68 | 103.22±1.60 | 14.76±1.31 | 20.24±2.38 | 1.39±0.08 | 28.86±1.66 |
|  | COF | 106.37±3.05 | 56.59±3.10 | 159.20±7.95 | 64.30±1.55 | 14.27±1.13 | 2.11±0.10 | 45.33±3.02 |
| **SD** | CF | 599.87±39.08 | 15.79±2.30 | 207.94±69.71 | 16.77±2.52 | 25.37±2.57 | 4.50±1.09 | 40.03±8.90 |
|  | COF | 304.04±40.15 | 56.72±10.65 | 386.74±23.93 | 59.86±8.97 | 44.89±3.20 | 8.73±1.07 | 75.99±0.95 |
| **AH** | CF | 712.14±92.34 | 3.00±0.70 | 166.22±29.14 | 22.54±3.70 | 60.89±6.82 | 5.00±0.55 | 36.11±8.65 |
|  | COF | 493.05±54.05 | 18.68±2.19 | 464.82±208.38 | 32.24±2.48 | 62.93±7.65 | 6.22±0.18 | 57.34±3.14 |
| **HN** | CF | 457.57±37.52 | 3.78±0.69 | 423.98±47.02 | 38.57±10.91 | 37.43±4.19 | 2.87±0.24 | 32.42±8.56 |
|  | COF | 670.41±172.11 | 6.14±1.12 | 202.81±49.73 | 25.56±4.09 | 64.11±11.31 | 9.18±0.48 | 51.02±8.91 |

The values are expressed as means ± S.E. JL: the long-term experiment conducted in Jilin Province of China; SD: the long-term experiment conducted in Shandong Province of China; AH: the long-term experiment conducted in Anhui Province of China; HN: the long-term experiment conducted in Hunan Province of China. CF means the treatment applied with only chemical fertilizer, and COF means the treatment applied with both organic and inorganic fertilizer.
